# Construction of a novel prognostic model for gastric cancer based on pharmacokinetics-related genes and comprehensive prognostic analysis

**DOI:** 10.3389/fgene.2025.1541401

**Published:** 2025-09-15

**Authors:** Yu Zhang, Kai Jia, Yuntong Guo, Xiaole Ma, Tian Yao, Feng Wu, He Huang

**Affiliations:** Department of Gastrointestinal Surgery, First Hospital of Shanxi Medical University, Taiyuan, Shanxi, China

**Keywords:** gastric cancer, ADME, prognosis genes, immune environment, prognostic model

## Abstract

**Background:**

Absorption, distribution, metabolism, and excretion of drugs-related genes (ADMERGs), pivotal in cancer occurrence, development, and chemotherapy resistance, lack investigation in gastric cancer (GC). Thus, this study aims to build a prognostic model for gastric cancer utilizing ADMERGs.

**Methods:**

The GC-related datasets, including TCGA-GC, GSE62254, GSE163558 and GSE13911, as well as 298 ADMERGs, were retrieved in this study. Prognostic risk models associated with ADME were developed utilizing univariate Cox analysis, followed by additional refinement using the least absolute shrinkage and selection operator (LASSO). The entire pool of gastric cancer (GC) patient samples was partitioned into high and low-risk categories, delineated by the median value of their respective risk scores. Within these two distinct groups, we conducted enrichment analysis, immune infiltration, and prognostic evaluation of ADME-related prognostic genes to uncover their molecular mechanisms in GC. The construction of ceRNA regulatory networks was undertaken to analyse the prognostic gene regulatory mechanisms. We analyzed single-cell data in GC to investigate the mechanisms driving its onset and progression at the cellular level. Additionally, we validated the expression trends of prognostic genes in clinical samples using RT-qPCR.

**Results:**

A prognostic model for GC was established and validated, comprising five genes (*UGT1A1, ADH4, ADH1B, CYP19A1,* and *GPX3*). The levels of infiltration of 21 immune cells exhibited significant disparities between the two risk groups, such as central memory CD4 T cells, activated B cells, and mast cells. There was a notable positive correlation between the risk scores and mast cells and plasmacytoid dendritic cells. In the high-risk group, the TIDE scores were heightened. The single-cell dataset showed significant under-expression of *ADH1B, ADH4, CYP19A1*, and *GPX3* in tumor samples. Finally, RT-qPCR showed that all the prognostic genes except for ADH4 were under-expressed in tumor tissues.

**Conclusion:**

We have developed and validated an innovative prognostic risk model for GC, revealing that elevated ADMERGs risk scores are indicative of unfavorable prognosis and diminished immunotherapy response. These findings furnish molecular evidence regarding the participation of ADMERGs in modulating the immune microenvironment and therapeutic responsiveness in GC.

## 1 Introduction

Gastric cancer (GC) is a significant worldwide health challenge, being the fifth most prevalent form of cancer and the third leading cause of cancer-related fatalities. The disease presents an urgent threat to both public health and global economies due to its high occurrence and death rates (De Martel et al., 2020; Rawla and Barsouk, 2019). The GC is commonly correlated with environmental variables such as infection by *Helicobacter pylori*, dietary choices, and lifestyle, but the diffuse type tends to have a more aggressive clinical progression and is typically associated with genetic factors (Smyth et al., 2020). The clinical manifestation of GC exhibits considerable heterogeneity, with symptoms that span from vague dyspepsia to more severe indications, hence adding complexity to the management and prognosis (Yang et al., 2021). Advancements in the understanding of the development of GC have resulted in better methods for diagnosis and treatment. However, there are still considerable obstacles to overcome.

ADME, which stands for absorption, distribution, metabolism, and excretion, encompasses the fundamental processes in pharmacokinetics (Furuta et al., 2005). It outlines the path a medication takes from its introduction into the body to its removal. The latest progress in the research of ADME related genes (ADMERGs) has emphasized their vital functions in the regulation of drug-metabolizing enzymes, transport proteins, and nuclear receptors, hence exerting a major impact on these pharmacokinetic processes (Aitken et al., 2008; Fletcher et al., 2010). ADMERGs have been found to have important roles in the genesis and progression of GC. Genetic variations in specific metabolic enzyme genes and transport protein genes can have an influence on the way chemotherapeutic medications are metabolized and distributed in the body. This, in turn, can have an impact on the effectiveness of treatment and the development of drug resistance. Furthermore, the presence of ADMERGs can impact the availability and toxicity of drugs, ultimately affecting how patients respond to treatment and their overall prognosis (Rodrigues, 2022). While research has revealed certain functions of ADMERGs in GC, the specific methods by which they operate are still not well understood. Hence, it is imperative to do additional study on the correlation between ADMERGs and the prognosis of GC to develop more effective tailored treatments.

In this paper, differentially expressed genes (DEGs) associated with ADME were found using the TCGA-GC dataset from the TCGA database, and candidate genes were obtained by taking intersections with ADEMRGs. Prognostic genes closely associated with GC outcome were identified by univariate Cox regression analysis and Least Absolute Shrinkage and Selection Operator (LASSO) regression. Subsequently, a predictive risk model was created using prognostic genes to classify patients based on risk scores. In addition, a nomogram was created to accurately predict the prognosis of GC patients. To investigate the biological mechanisms of these prognostic genes, enrichment analysis, immune infiltration, drug sensitivity analysis, ceRNA network construction, and analysis of prognostic gene expression patterns at the single-cell level were also performed. The findings will provide novel insights into the molecular mechanisms behind GC and propose prospective targets for therapeutic intervention, as well as serve as a helpful reference for future research on the etiology and treatment strategies of GC.

## 2 Materials and methods

### 2.1 Data sources

For the development of a predictive model for GC, we accessed transcriptome data and corresponding clinical pathology data from The Cancer Genome Atlas (TCGA, http://portal.gdc.cancer.gov/). This dataset consists of 375 tumor samples and 32 normal samples and serves as the foundational training set, of these, 350 tumor samples had survival information. The datasets GSE62254 (GPL570) including 300 tumor tissue samples and GSE13911 including 69 samples were used as the validation set (Cristescu et al., 2015; Oh et al., 2018) and were obtained from Gene Expression Omnibus database (GEO, https://www.ncbi.nlm.nih.gov/geo/). A collection of 298 ADMERGs was sourced from the PharmaADME Consortium (http://www.pharmaadme.org) (Tang et al., 2022).

### 2.2 Identification and enrichment analysis of candidate genes

We employed the R package DESeq2 (version 1.34.0) to carry out differential expression analysis between tumor and normal samples within our training set. Genes that exhibited a |log_2_FoldChange| > 2 and an adj.P-value <0.05 were identified as significant and were selected based on these criteria (Love et al., 2014). To obtain candidate genes, the intersecting set of DEGs and ADMERGs was determined using the R package ggvenn (version 1.2.2). Enrichment analysis, which encompasses Gene Ontology (GO) and Kyoto Encyclopedia of Genes and Genomes (KEGG), was conducted using the R package clusterProfiler (version 3.18.1), with an adjusted p-value threshold of less than 0.05 (Yu et al., 2012). The Oncobox database was utilized to assess the KEGG pathway activation level of candidate genes, adj.P < 0.05 was used as the screening threshold.

### 2.3 A prognostic risk model linked to ADME was constructed and validated

The candidate genes associated with prognosis were identified using univariate Cox regression analyses, employing the glmnet package (version 4.1-2) (HR ≠ 1 & P < 0.05), using 10-fold cross-validation, GC samples were randomly and equally divided into 10 subsets. In each iteration, one subset was selected as the validation set, while the remaining nine subsets served as the training set. The LASSO-Cox model was fitted using the training set, and the Cox deviation of the model was evaluated on the validation set. After 10 iterations, the average deviations of all λ values on the validation sets were compared, and λ.min, which minimized the cross-validation error, was selected as the final regularization parameter. Finally, the optimal λ value was determined, and the final LASSO-Cox model was constructed using all training data to screen out key prognostic gene features. The proportional hazards (PH) assumption was then employed to verify these genes, using a p-value threshold of greater than 0.05. Subsequently, we utilized LASSO regression analysis from the R package glmnet to determine the final prognostic genes (Friedman et al., 2010). Subsequently, a risk score was calculated for each GC patient, which was based on the expression (expr) and risk coefficients (coef) of these prognostic genes, as follows:

$$\text{Riskscore}=\sum_{i=1}^{n}\text{coef}\left({\text{gene}}_{i}\right)*\text{expr}\left({\text{gene}}_{i}\right)$$

Patients with GC were categorized into either a high- or low-risk groups based on the median risk score. Additionally, KM curves (P < 0.05) were plotted according to the high and low risk groups using survminer (version 0.4.9) (Liu et al., 2021) to determine the difference in survival between the high and low risk groups. The receiver operating characteristic (ROC) curves (2, 3, 4, 5, 6 years) were generated utilizing the survival and survival ROC packages (version 1.0.3.1) (Maeser et al., 2021) to assess the model’s accuracy (AUC >0.6). In the validation set, the same method was employed. Subsequently, the cor function from the R package “stats” was employed to assess correlations between prognostic genes and established GC (gastric cancer) prognostic markers, including MSI (microsatellite instability), PD-L1 expression, and HER2 status, using both Spearman and Pearson correlation analyses.

### 2.4 Correlation between clinical features and risk score

The utilization of the Wilcoxon rank-sum test (P < 0.05) allowed for the evaluation of variations in risk scores among distinct clinical subgroups and the investigation of patient distribution into high and low-risk groups across these subgroups, taking into account clinical factors including Age, Gender, T/N/M stage, and Stage.

### 2.5 Establishment and validation of a nomogram

Via univariate and multivariate Cox regression analyses utilizing the R package survival (P > 0.05; HR≠1), identified independent prognostic factors influencing GC, with the results confirmed by the PH hypothesis test (P > 0.05). Subsequently, a prognostic nomogram was developed employing the rms package (version 6.2-0) (Luo et al., 2024). This was followed by the generation of decision curve analysis (DCA), calibration curves and ROC curves to gauge the accuracy and reliability of the nomogram.

### 2.6 Gene set enrichment analysis (GSEA) and gene set variation analysis (GSVA)

Across risk groups, GSEA was executed via the R package clusterProfiler, employing c2.cp.kegg.V7.0.symbols.gmt as the reference gene set. Meanwhile, the analysis applied a stringent significance threshold of P < 0.05 and a False Discovery Rate (FDR) of <0.25 for the screening process. GSVA was performed utilizing the R package “GSVA” (version 1.42.0) (Hänzelmann et al., 2013). To solidify the findings, a Spearman correlation analysis was meticulously conducted to elucidate the intricate relationship between clinical manifestations, risk scores, and biological pathways.

### 2.7 Immune cell infiltration analysis

In the training set, employing single-sample GSEA (ssGSEA) to assess the abundance of 28 immune infiltrating cells and 2 stromal cells. Following this, a stringent Wilcoxon test (with a significance threshold of P < 0.05) was applied with a significance threshold of P < 0.05 to identify significant differences in the abundance of immune infiltrating cells between two groups. Furthermore, to delve deeper into the association between risk score and differentially infiltrating cells, a Spearman correlation analysis was conducted (with |R| > 0.4 and P < 0.05). Next, the cor function from the R package stats was used to perform Pearson correlation analysis between prognostic genes and differentially infiltrated immune cell subsets.

### 2.8 Immunotherapy and prediction of chemotherapy response

We assessed the varying expression of 15 m6A RNA methylation genes and 24 immune checkpoint genes between two risk groups, aiming to forecast the potential effectiveness of immune checkpoint blockade (ICB) in patients with different risk levels (P < 0.05). Additionally, we calculated the Tumor Immune Dysfunction and Exclusion (TIDE) score for each GC patient using the TIDE database (http://tide.dfci.harvard.edu/). To further predict the clinical response to chemotherapy, we evaluated the sensitivity of drugs using the R package pRRophetic (version 0.5) (Geeleher et al., 2014) (P < 0.05). Next, the cor function and cor.test significance test built into R language were used, and the Spearman correlation analysis method was adopted to calculate the correlation between prognostic genes and immune checkpoint genes (p < 0.05).

### 2.9 The ceRNA regulatory network analysis

To investigate the molecular regulatory mechanisms between high-risk and low-risk groups, in the training set, utilizing DESeq2 (v.1.34.0) (Love et al., 2014) to identify differentially expressed miRNAs (miRNA1), and lncRNAs (lncRNA1) between GC high-risk group and low-risk group (|log_2_FoldChange|>1 and adj.P.value<0.05). Additionally, miRDB (http://www.mirdb.org/miRDB/policy.html) databases were utilized to predict prognostic genes miRNAs (miRNA2). Key miRNAs were obtained by intersecting miRNA1 and miRNA2 using the package ggvenn. Furthermore, upstream lncRNAs (lncRNA2) of key miRNAs were predicted using the miRNet(https://www.mirnet.ca) databases. Key lncRNAs were obtained by intersecting lncRNA1 and lncRNA2 with the ggvenn package. Finally, we constructed a ceRNA regulatory network using these key molecules.

### 2.10 The scRNA-seq data analysis

The scRNA-seq data underwent quality control measures implemented through the R package Seurat (version 4.1.0) (Hao et al., 2021). Exclusion criteria as follows: cells with less than 200 genes expressed, genes detected in fewer than 3 cells, and cells with a mitochondrial gene proportion above 20%. Additionally, cells expressing less than 101 or more than 6000 genes were filtered out. Subsequently, the Seurat package’s integrated was then performed to correct for batch effects. To reduce dimensionality and cluster cells, we used the RunPCA function with the t-SNE algorithm. Following clustering, we used the FindAllMarkers function and the singleR package (Aran et al., 2019) to annotate each cell. After annotation to obtain the cell types, a bar stacking plot was drawn to show the distribution of different cell types in tumor and normal samples, as well as UMAP plots and prognostic genes in each cell type distribution and expression in each cell type. Finally, the Wilcoxon test was used to compare the differences in the expression of prognostic genes between gastric cancer tumor tissue samples and normal samples, as well as the expression of biomarkers in annotated cell types.

### 2.11 Reverse transcription quantitative PCR (RT-qPCR)

From the Department of gastrointestinal surgery in First Hospital of Shanxi Medical University, we collected the tumor (n = 5) and para-carcinoma tissues (n = 5) of patients with GC. All donor patients provided and signed off on the informed consent, which was approved by the Ethics Committee of our hospital (Ethics Review No:KYLL-2024-075). The total RNA of all samples was isolated in terms of TRIzol Reagent (Ambion, Shanghai, China). Subsequently, total RNA was used to reverse transcription via the SweScript First Strand cDNA synthesis kit (Servicebio, Wuhan, China). Then, the primers of prognostic genes were shown in Supplementary Table S1. The qPCR was proceeded using the Universal Blue SYBR Green qPCR Master Mix (Servicebio, Wuhan, China) on the CFX96TM PCR System (BIO-RAD, U.S.A.). The relative expression of these prognostic genes was calculated based on the 2^−ΔΔCT^ method (Rao et al., 2013), with an endogenous control GAPDH.

### 2.12 Statistical analysis

All bioinformatics analyses were performed using the R language (version 4.2.2). P < 0.05 was considered meaningful and significant.

## 3 Results

### 3.1 Identification and enrichment analysis of candidate genes

**FIGURE 1:**
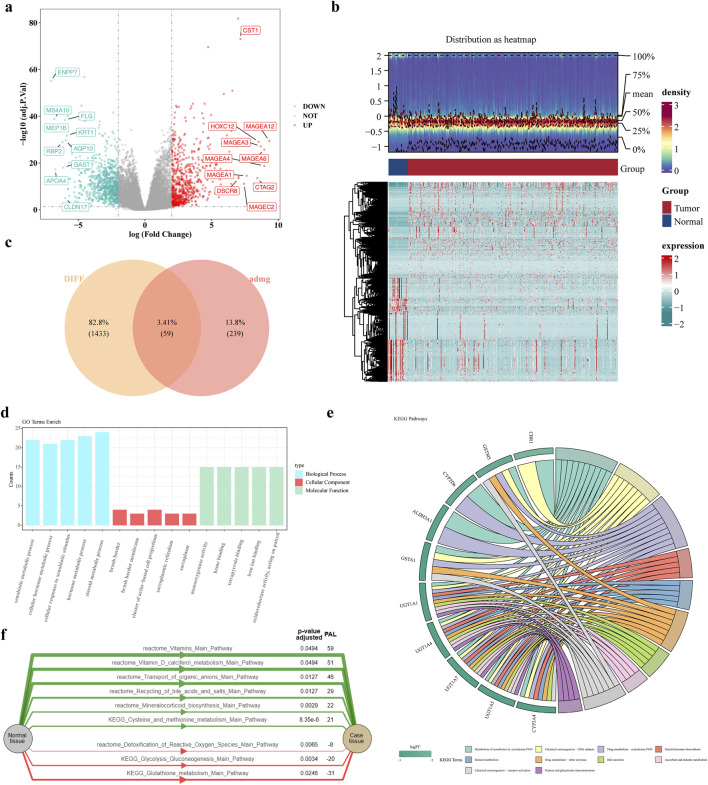
Identification and enrichment analysis of candidate genes. **(a,b)** Volcano and heat maps of differentially expressed genes **(c)** Venn diagram of candidate genes **(d)** GO enrichment results of candidate genes **(e)** KEGG enrichment results **(f)** Activation level analysis of KEGG pathway. The green color of the graph represents the activation pathway and the red color represents the inhibition pathway.

A total of 1,492 DEGs were identified, with 683 upregulated and 809 downregulated in the GC patients (Figures 1a,b). Moreover, 59 candidate gene were obtained by intersecting ADMERGs and DEGs using a Venn diagram (Figure 1c). Following this, candidate genes GO enrichment analysis results showed, the enriched biological process categories included cellular hormone metabolic process, cellular response to xenobiotic stimulus, hormone metabolic process, xenobiotic metabolic process, and steroid metabolic process. The primary enriched cellular component categories included brush border membrane, sarcoplasmic reticulum, brush border, sarcoplasm, cluster of actin-based cell projections. The primary enriched molecular function categories included monooxygenase activity, tetrapyrrole binding, and so on (Figure 1d). The results of the KEGG enrichment analysis indicated that the candidate genes were predominantly enriched in metabolic pathways, metabolism of xenobiotics by chemical carcinogenesis-DNA adducts, drug metabolism-cytochrome P450, retinol metabolism, and steroid hormone biosynthesis (Figure 1e). Pathway activation level analysis showed that six pathways exhibited significant activation in GC samples, include vitamin, transport of organic anions, recycling of bile acids and salts, mineralocorticoid biosynthesis, cysteine and methionine metabolism main pathway. And three pathways were significantly inhibited, including detoxification of reactive oxygen species, glycolysis gluconeogenesis, glutathione metabolism main pathway (Figure 1f).

### 3.2 Construction of a prognostic models related to ADME

**FIGURE 2:**
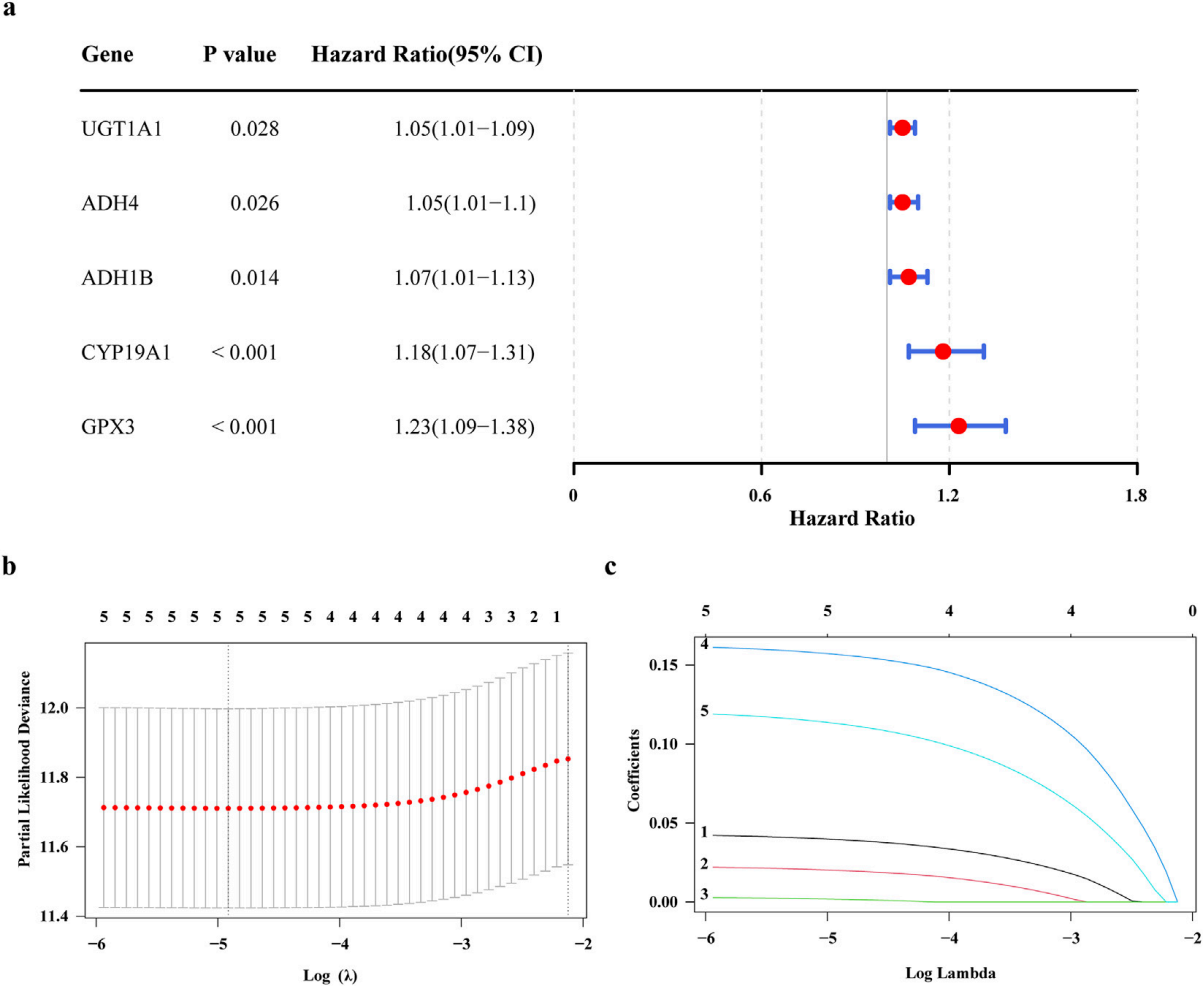
Construction of a prognostic models related to ADME. **(a)** Forest plot of one-way Cox regression analysis **(b)** Cross-validation of prognostic gene LASSO regression analysis **(c)** prognostic gene LASSO regression analysis.

To obtain genes associated with GC prognosis in the training set, using the univariate Cox regression analysis and verified by the PH hypothesis test, resulting in the screening out of 5 genes associated with survival (Figure 2a). Subsequently, we utilized the LASSO for further refinement, resulting in the identification of 5 prognostic genes: UGT1A1, ADH4, ADH1B, CYP19A1, GPX3 (Figures 2b,c). Analyze the correlation between these prognostic genes and known GC prognostic markers such as MSI, PD-L1, HER2. The results showed that UGT1A1, ADH4 were significantly negatively correlated with PD-L1, and UGT1A1, GPX3, ADH1B were significantly correlated with HER2 (Supplementary Figure S1). Among them, UGT1A1 was significantly positively correlated with HER2, GPX3, ADH1B were significantly negatively correlated with HER2, and GPX3, ADH1B, ADH4 were significantly negatively correlated with MSI. The ADMERGs plays a dual role in metabolic reprogramming and immune microenvironment regulation, significantly affecting key prognostic markers of gastric cancer (PD-L1, HER2, MSI). UGT1A1, GPX3, ADH1B, and ADH4 can serve as cross pathway regulatory nodes and can be integrated into a “metabolic immune prognostic model” in the future to optimize precision treatment strategies for gastric cancer.

### 3.3 Assessment of prognostic characteristics

**FIGURE 3:**
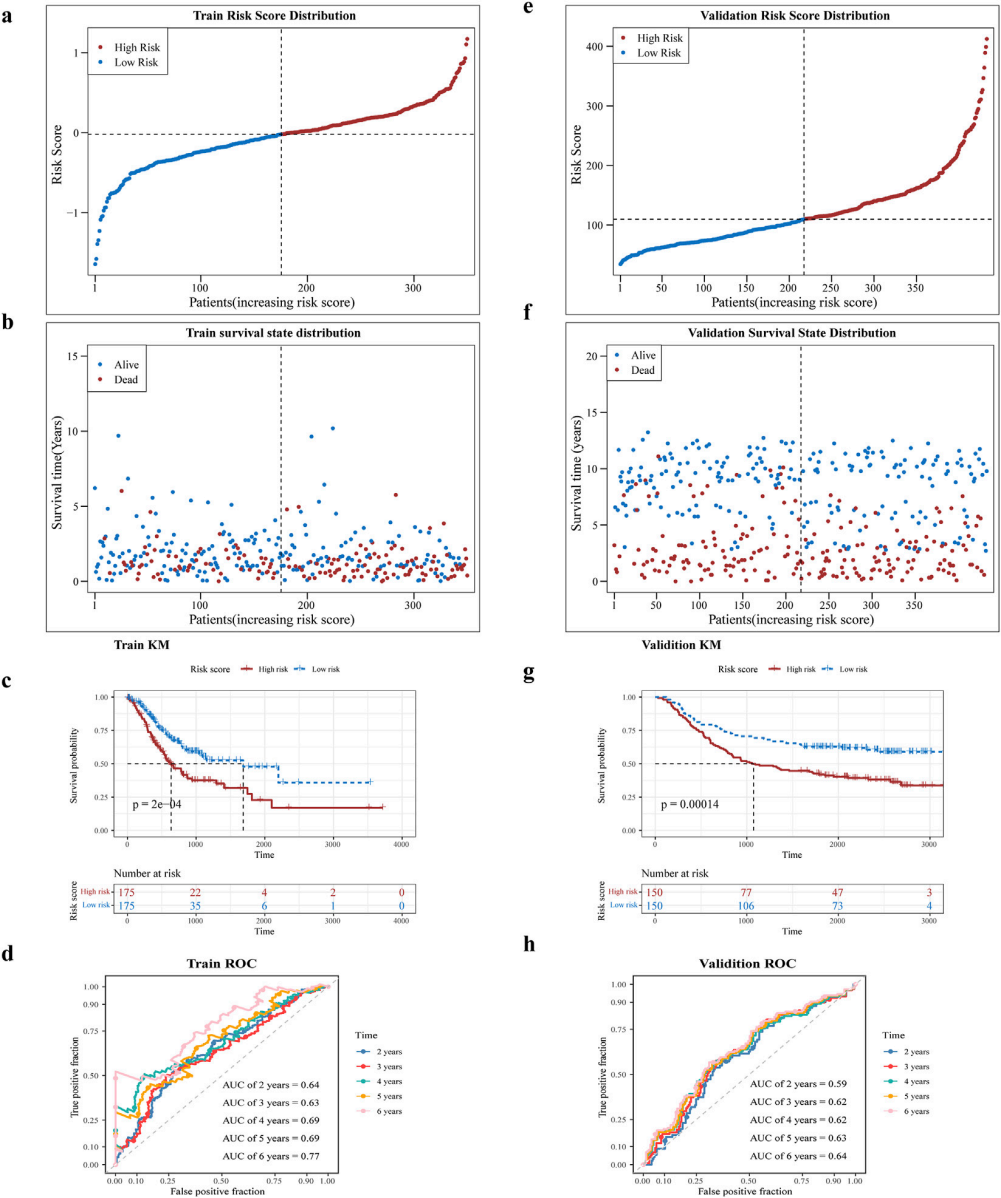
Assessment of prognostic characteristics. **(a,b)** Risk profiles of the training set GC patients in high and low risk subgroups will and scatter plots. **(c)** Prognostic gene K-M survival analysis. The horizontal axis is the total survival time (days) and the vertical axis is the survival probability; red color represents the high-risk group and blue color represents the low-risk group. **(d)** ROC curves of training set GC patients at 2, 3, 4, 5, and 6 years **(e,f)** Risk curves and scatter plots of high- and low-risk subgroups of validation set GSE62254 samples. **(g)** K-M survival analysis of high- and low-risk subgroups of validation set GSE62254 samples **(h)** ROC curves of validation set GSE62254 patients at 2, 3, 4, 5, and 6 years.

Risk scores were computed for all patient samples in the training set, leading to their classification into either a high-risk or low-risk group, rested on the median value. The high-risk group exhibited decreased survival rates and less survival times in contrast to the low-risk group. Additionally, a notable rise in the number of deaths was observed with increasing risk scores among the samples (Figures 3a,b). Afterward, KM curve showed significantly lower survival in the high-risk group than in the low-risk group, as illustrated in Figure 3c (P < 0.005). In the training set, the area under curve (AUC) values for the risk score were 0.64, 0.63, 0.69, 0.69, and 0.77 at 2, 3, 4, 5, and 6 years, respectively, as presented in Figure 3d. The same method was employed to verify the model’s accuracy and applicability in the test set, with the risk survival status of GC patients was also displayed (Figures 3e–g). The AUC values were 0.59, 0.62, 0.62, 0.63, and 0.64 at 2, 3, 4, 5 and 6 years, in the test set (Figure 3h), suggesting that the risk model has good predictive power.

### 3.4 Correlation of risk score and other clinicopathological features

**FIGURE 4:**
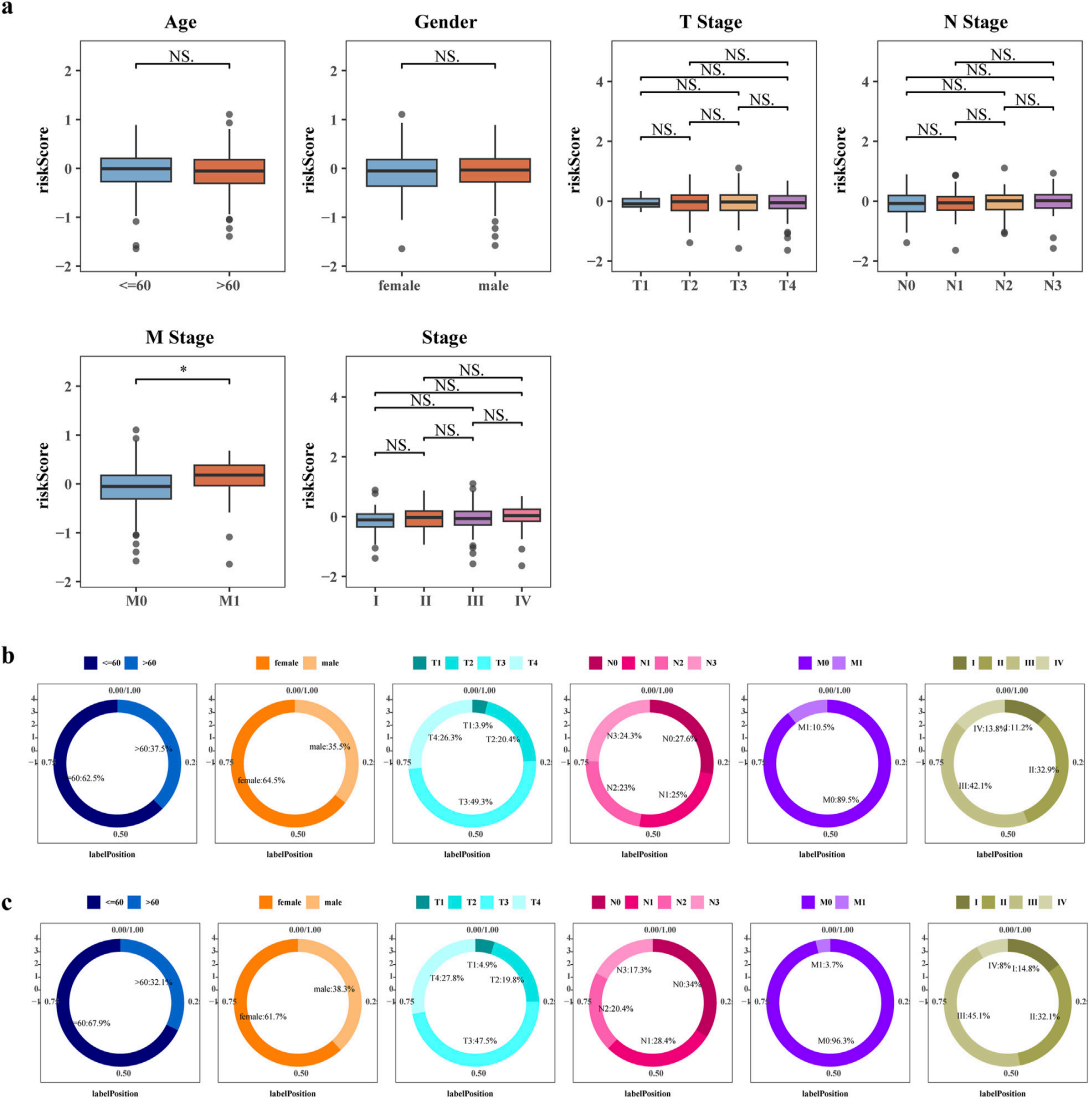
Correlation of risk score and other clinicopathological features. **(a)** Boxplot of differences in risk scores between clinical subgroups. Differences in the distribution of patients in the high risk groups **(b)** and low risk groups **(c)** between clinical subgroups.

To investigate the involvement of risk score in GC, we assessed their association with clinical characteristics. Utilizing the clinical data obtained from TCGA, GC patients were categorized into distinct subgroups. Our analysis unveiled noteworthy disparities in risk scores between the M0 and M1 groups (Figures 4a–c).

### 3.5 The nomogram was constructed and validated

**FIGURE 5:**
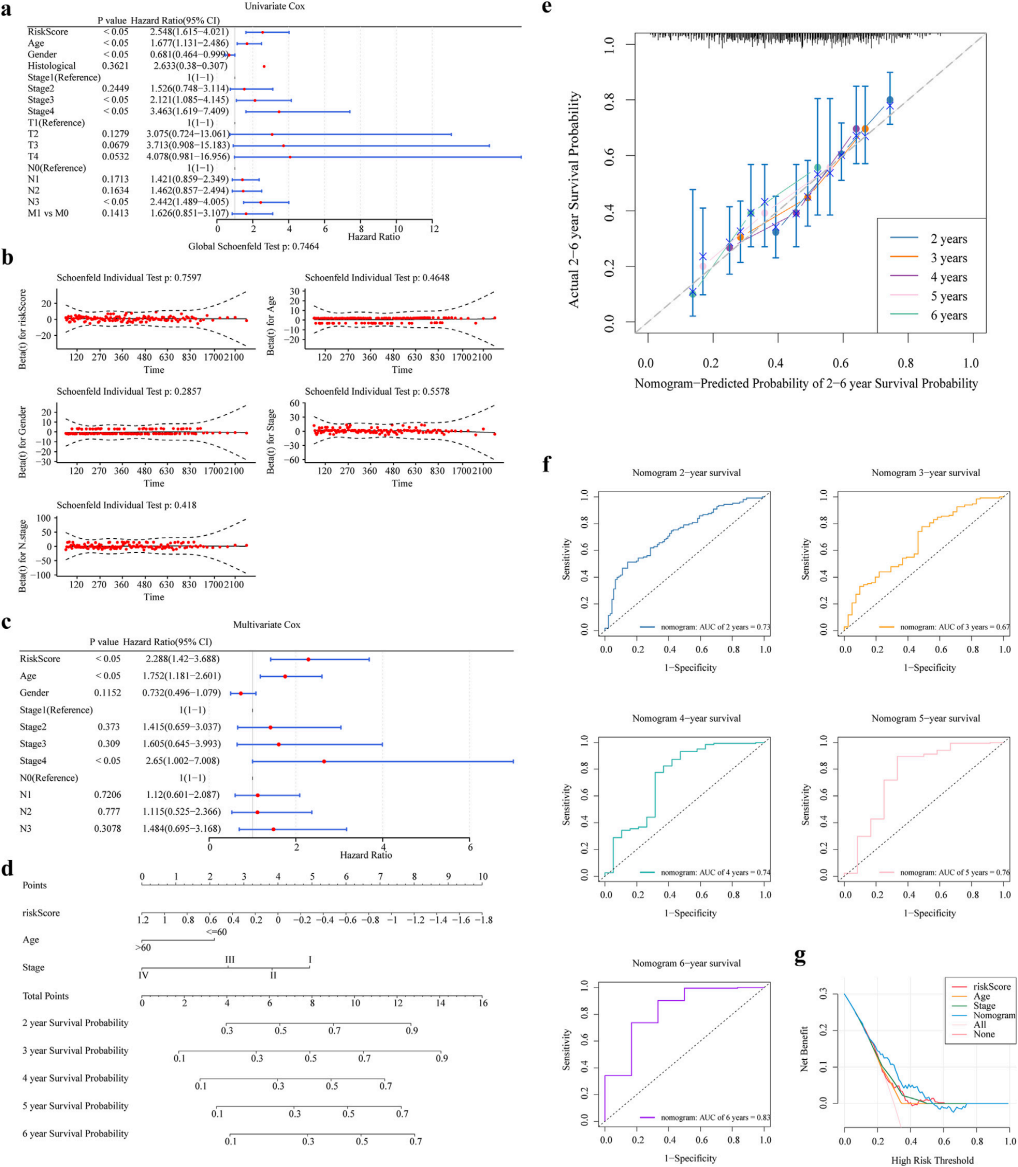
The nomogram was constructed and validated. **(a,b)** One-way Cox Analysis and PH Hypothesis Test **(c)** Results of Multifactor Cox Analysis **(d)** Nomogram Columns **(e)** Columns 2, 3, 4, 5, and 6 Years Calibration Curves. **(f,g)** Columns 2, 3, 4, 5, and 6 Years ROC and DCA Curves.

Risk score, age, and stage were significant independent predictors of patient outcome (P < 0.05) (Figures 5a–c). We created a nomogram based on these independent prognostic factors (Figure 5d). Figure 5e exhibits the calibration curves for the nomogram, illustrating the probability of Overall Survival (OS) at 2, 3, 4, 5, and 6 years. In addition, the ROC and DCA curve was generated to evaluate the nomogram’s accuracy (Figures 5f,g). The AUC values for the nomogram surpassed 0.6 across 2, 3, 4, 5, and 6 years, suggesting a positive predictive performance. All AUC values were greater than 0.6 and the decision curve of the nomogram was higher than any of the independent prognostic factors, suggesting that a nomogram combining risk scores with clinical aspects is valuable for the diagnosis of GC.

### 3.6 GSEA and GSVA analysis

**FIGURE 6:**
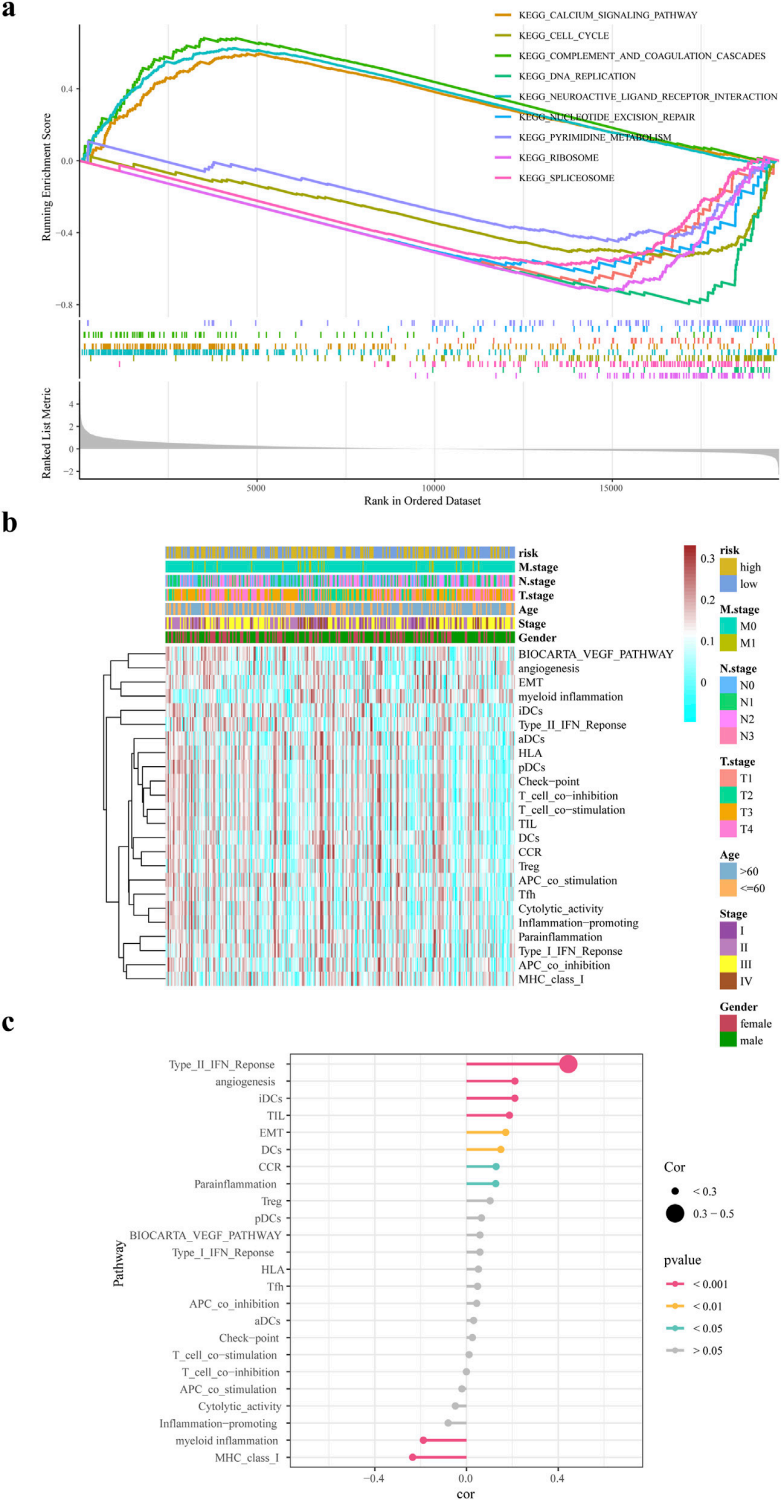
GSEA and GSVA analysis. **(a)** GSEA enrichment analysis of differential genes in high and low risk groups **(b)** ssGSEA biological pathway analysis **(c)** Correlation analysis of risk scores and biological pathways.

In this study, we identified 48 pathways that were noticeably abundant between the two groups. The high-risk group exhibited significant enrichment in pathways such as the calcium signaling pathway and complement and coagulation cascade. Conversely, the low-risk group showed significant enrichment in pathways including DNA replication, spliceosome, ribosome, nucleotide excision repair, and the cell cycle, (Figure 6a). Figure 6b illustrates the correlation among risk scores, clinical features, and typical biological pathways. This finding suggested a substantial positive correlation between the risk score and the type II interferon response, as shown in Figure 6c.

### 3.7 Immune microenvironment analysis

**FIGURE 7:**
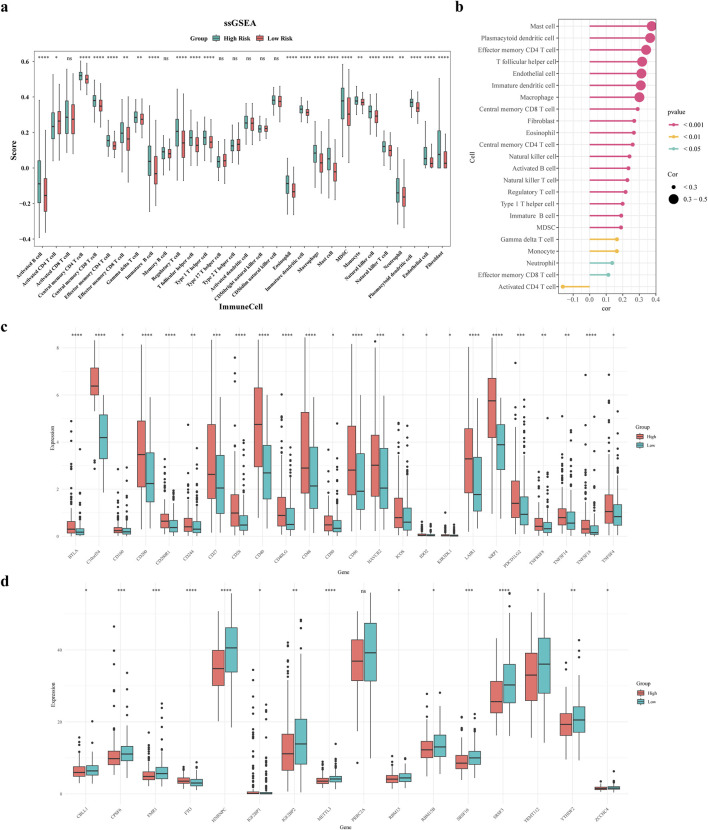
Immune microenvironment analysis. **(a)** Differential violin plots of immune infiltrating cells in samples from high and low risk groups **(b)** Correlation analysis between risk scores and differential immune cells **(c)** Differential violin plots of immune checkpoints in samples from high and low risk groups **(d)** Differential violin plots of m6A-related genes in samples from high and low risk groups.

The findings revealed significant differences in abundance for 21 immune cells and 2 stromal cells (fibroblasts and endothelial cells) between the two risk groups, for instance effector memory CD8 T cell, activated B cell, central memory CD4 T cell, central memory CD8 T cell, etc., (Figure 7a). In addition, a positive relationship was found between the risk score and the abundance of cell types including mast cells, plasmacytoid dendritic cells, and effector memory CD4 T cells (Figure 7b). Subsequently, correlation analysis was conducted between prognostic genes and differential immune cells. The results showed that ADH1B, GPX3, and most immune cells had strong positive correlations, while UGT1A1, ADH4, CYP19A1 had weak correlations with some immune cells (Supplementary Figure S2).

Figure 7c demonstrates substantial variances in the expression levels of 24 immune checkpoints among the two risk groups, and overexpressed in the high-risk group. Comparing m6A-related genes (m6A-RGs), 15 differentially expressed genes were identified. Except for FTO and IGF2BP1, the rest of the differential m6A genes had increased expression in the low-risk group (Figure 7d). Subsequently, we analyzed correlations between prognostic and immune checkpoint genes. Results showed: UGT1A1 was significantly negatively correlated with PDCD1, CD274, PDCD1LG2, CTLA4, and LAG3; ADH4 was significantly negatively correlated with CD274; ADH1B was significantly positively correlated with TIGIT and PDCD1LG2, negatively with CD274; CYP19A1 was significantly positively correlated with HAVCR2, negatively with LAG3; GPX3 was significantly positively correlated with HAVCR2, LAG3, PDCD1LG2, and PDCD1 (Supplementary Figure S3). This indicates that these prognostic genes are deeply involved in the balance between the activity and inhibition of the immune microenvironment, providing new strategies for precise stratification and targeted metabolic sensitization in tumor immunotherapy.

### 3.8 Immunotherapy and prediction of chemotherapy response

**FIGURE 8:**
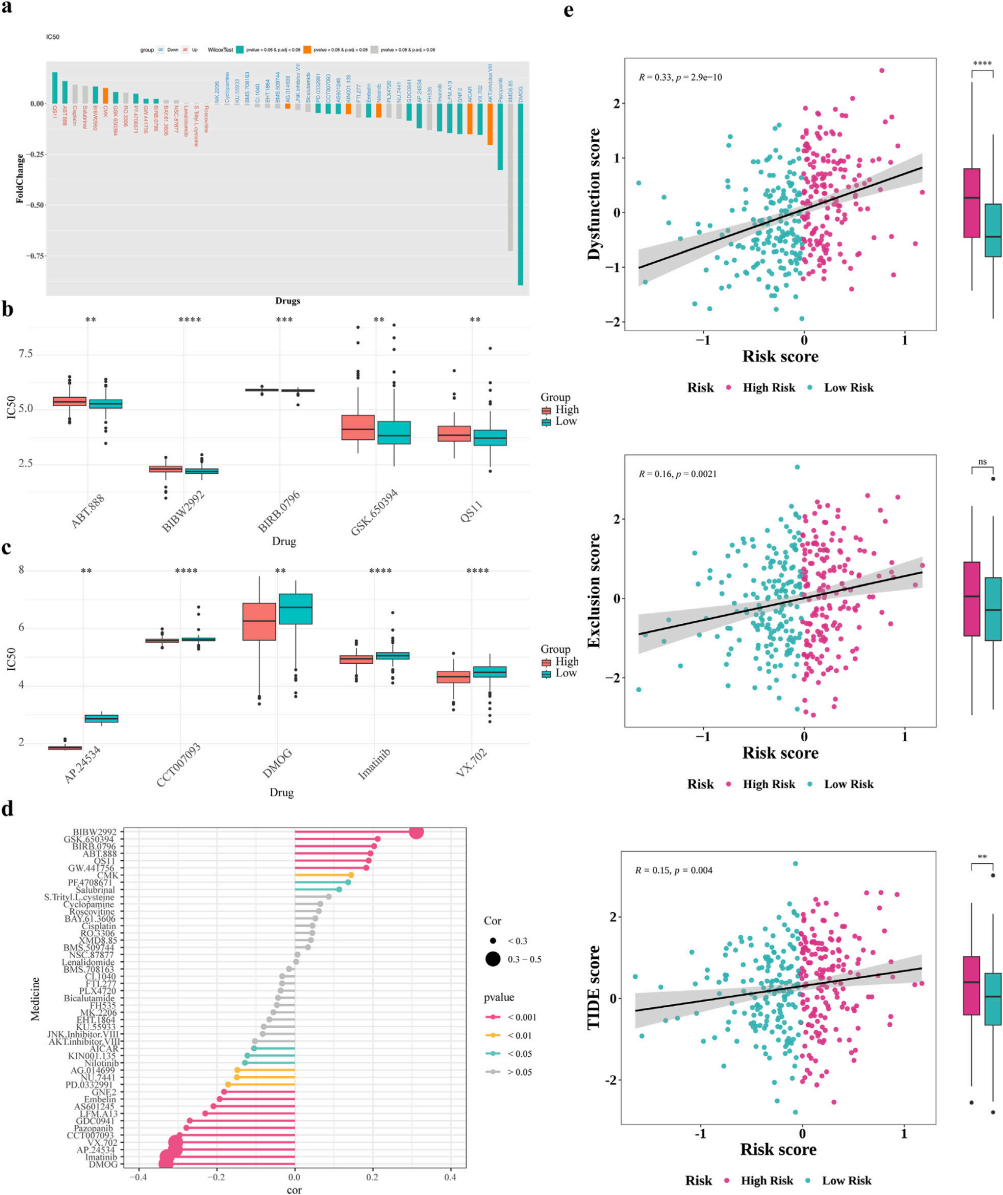
Immunotherapy and prediction of chemotherapy response. **(a)** Drug sensitivity analysis of patients in the high and low risk groups **(b,c)** Differences in top5 positive and negative correlation drug IC50 **(d)** Correlation analysis of drug IC50 values with risk scores **(e)** Differences in T-cell dysfunction and exclusion scores and TIDE scores of patients in the high and low risk groups.

This research aimed to explore the utility of the risk score in informing treatment strategy decisions for patients. We found that the low-risk group demonstrated enhanced responsiveness to the drugs QS11 and ABT.888, whereas the high-risk category showed increased sensitivity for DMOG and VX.702 (P < 0.05) (Figures 8a–c). The Spearman association analysis demonstrated a positive correlation between BIBW2992 and the risk score, as well as a negative correlation with DMOG, Imatinib, AP.24534, and VX.702. (Figure 8d). Besides, patients in the high-risk group revealed significantly higher Dysfunction and TIDE scores compared to those in the low-risk group (Figure 8e).

### 3.9 The ceRNA regulatory network analysis

**FIGURE 9:**
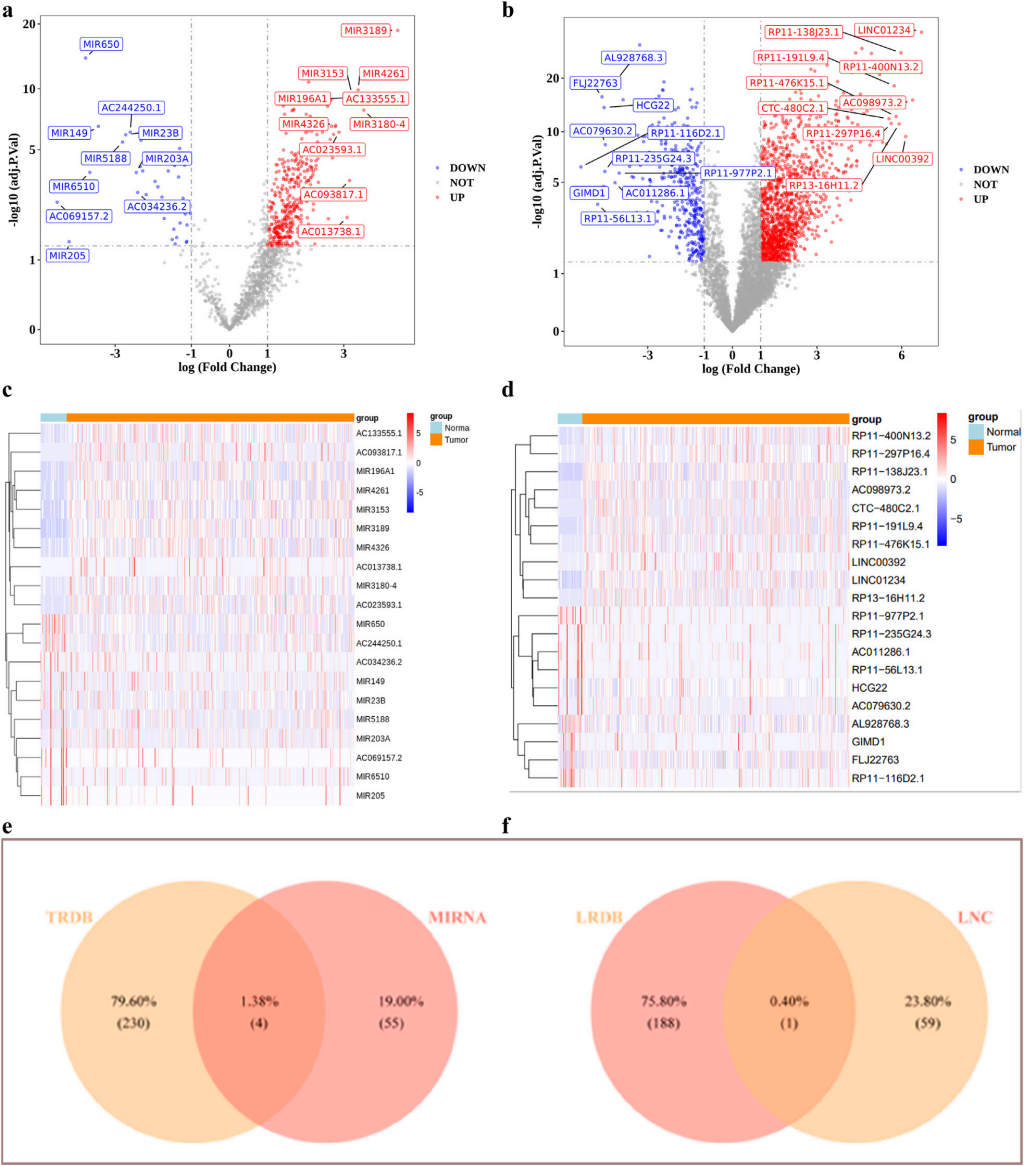
The ceRNA regulatory network analysis. **(a–d)** Volcano and heat maps of differentially expressed miRNAs and lnRNAs **(e,f)** Identification of targeted miRNAs, targeted lncRNAs.

In the training set, DESeq2 identified differentially expressed miRNA1 and lncRNA1 between GC high-risk group and low-risk group (Figures 9a–d). Using miRDB databases were predicted miRNA2, the Venn diagram illustrated the identification of four key miRNAs, mir590, mir7152, mir4420 and mir5000 were obtained (Figure 9e). The lncRNA2 of the key miRNAs was predicted using the miRNet databases, lncRNA1 and lncRNA2 were intersected to obtain a key lncRNA (MIR100HG) (Figure 9f). Among them, mir590, mir7152, mir4420 and mir5000 were significantly upregulated in the GC high-risk group (P < 0.05). Additionally, MIR100HG lncRNA was also significantly upregulated in the GC high-risk group (P < 0.05) (Supplementary Tables S2, S3).

### 3.10 scRNA-seq analysis

**FIGURE 10:**
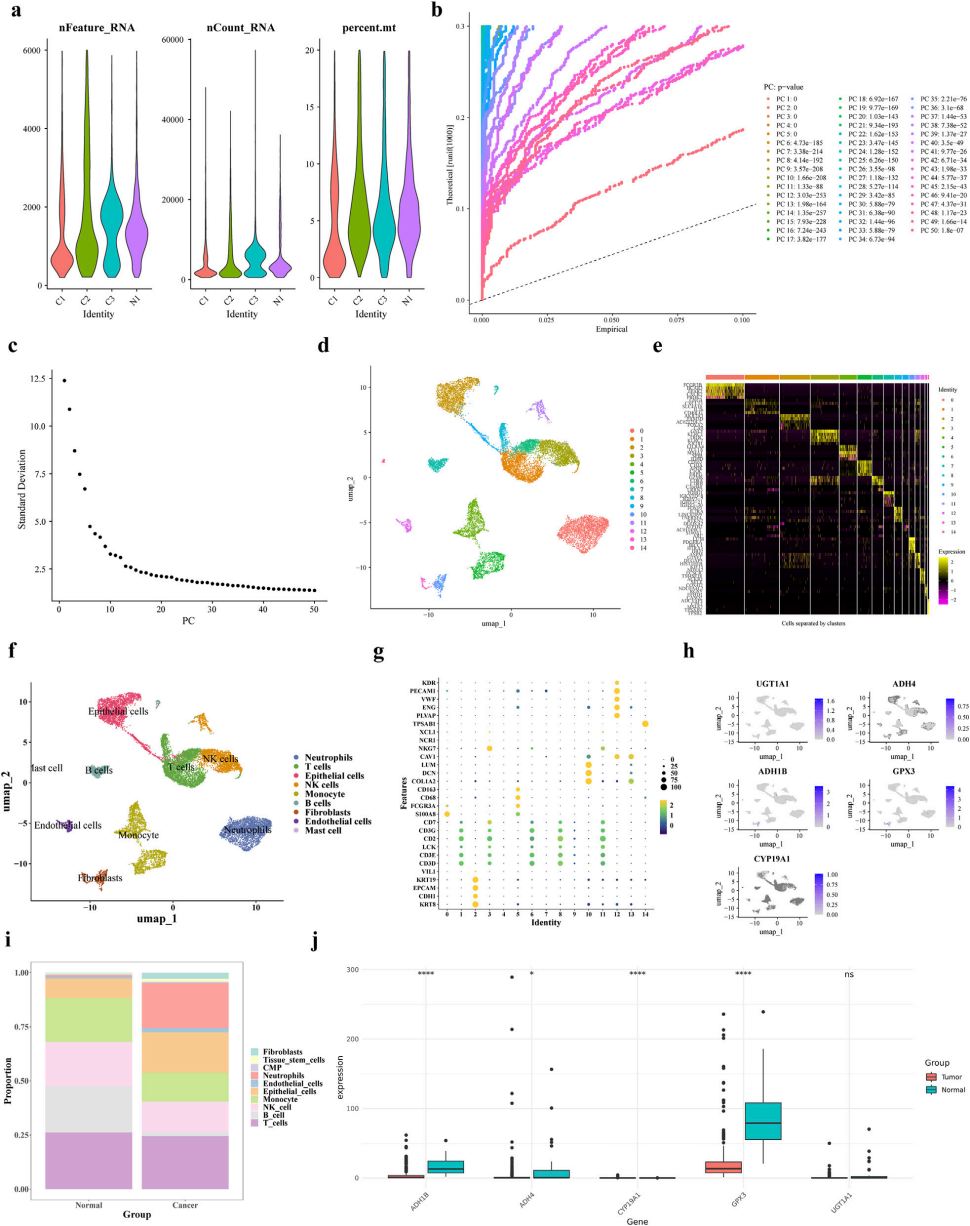
scRNA-seq analysis. **(a)** Results after QC of single-cell data **(b,c)** PCA principal component and fragmentation plots **(d)** UMAP clustering plots **(e)** Expression status of specific highly expressed genes in each cell population **(f)** Cellular annotation analysis **(g)** Expression levels of marker genes in each cell population obtained by the annotation **(h,i)** Gene distribution of prognostic genes in each cell type and bar stacking plots **(j)** Prognostic gene expression in training set gene expression in the training set.

The scRNA-seq data were processed using the R package “Seurat” for filtering. Then calculated nFeature-RNA, nCount-RNA and percent.mt (Figure 10a). The top 50 principal components with statistical significance from the PCA analysis were chosen for further analysis using UMAP the clustering analysis identified 15 distinct cell clusters (Figures 10b–d). We annotated 9 cell clusters based on marker genes from the CellMarker database and the R package “singleR”: T cells (CD3D, CD3E, LCK, CD2, CD3G, CD7), B cells (identified by SingleR auxiliary comments), Monocytes, NK cells, intermediate granule cells, endothelial cells, fibroblastic vascular cells, epithelial cells and mast cells (TPSAB1) (Figures 10e–g).

Subsequently, we conducted an analysis of each cell type proportion in the GC samples, as illustrated in Figures 10h,i. The T cells proportion was the largest in both tumor and control groups, while fibroblasts had the lowest proportion. In tumor tissues, neutrophil proportion was higher and B cells and T cells were lower compared to normal cells. The levels of expression for ADH1B, ADH4, CYP19A1, and GPX3 were notably decreased in tumor samples from the single-cell dataset, as depicted in Figure 10j. Subsequently, the expression levels of prognostic genes were compared and validated using the GSE13911 dataset, and the results showed that the expression trends of four prognostic genes, ADH1B, ADH4, CYP19A1, and GPX3, were consistent with them (Supplementary Figure S4). The Wilcoxon test was used to compare the expression of prognostic genes between GC tumor tissues and control samples in the TCGA training set and GSE13911 dataset. Results showed that four prognostic genes (ADH1B, ADH4, UGT1A1, and GPX3) exhibited significantly lower expression in tumor samples compared to normal samples (Supplementary Figure S5a). However, CYP19A1 showed upregulated expression in the TCGA training set but downregulated expression in the GSE13911 validation set, which might be attributed to differences in dataset characteristics or sample heterogeneity (Supplementary Figure S5b). Additionally, we analyzed the differential expression of prognostic genes across distinct cell types. As shown in Supplementary Figures S5a,b, GPX3 and ADH1B were more highly expressed in Stromal cells compared to other cell types. The Wilcoxon test was used to compare the differential expression of prognostic genes in annotated cell types between disease and normal physiological conditions. UGT1A1 showed significant expression differences between disease and normal conditions in T cells and Monocytes; ADH4 exhibited significant differences in Monocytes, B cells, Epithelial_cells, and Stromal cells; GPX3 showed significant variations in T_cells, Plasma, Epithelial_cells, and Stromal cells; ADH1B had significant differences in Plasma, Stromal cells, and Epithelial_cells; and CYP19A1 displayed significant expression differences in Stromal cells between disease and normal conditions. (Supplementary Figures S5c–g).

### 3.11 Expression level verification of prognostic genes

**FIGURE 11:**
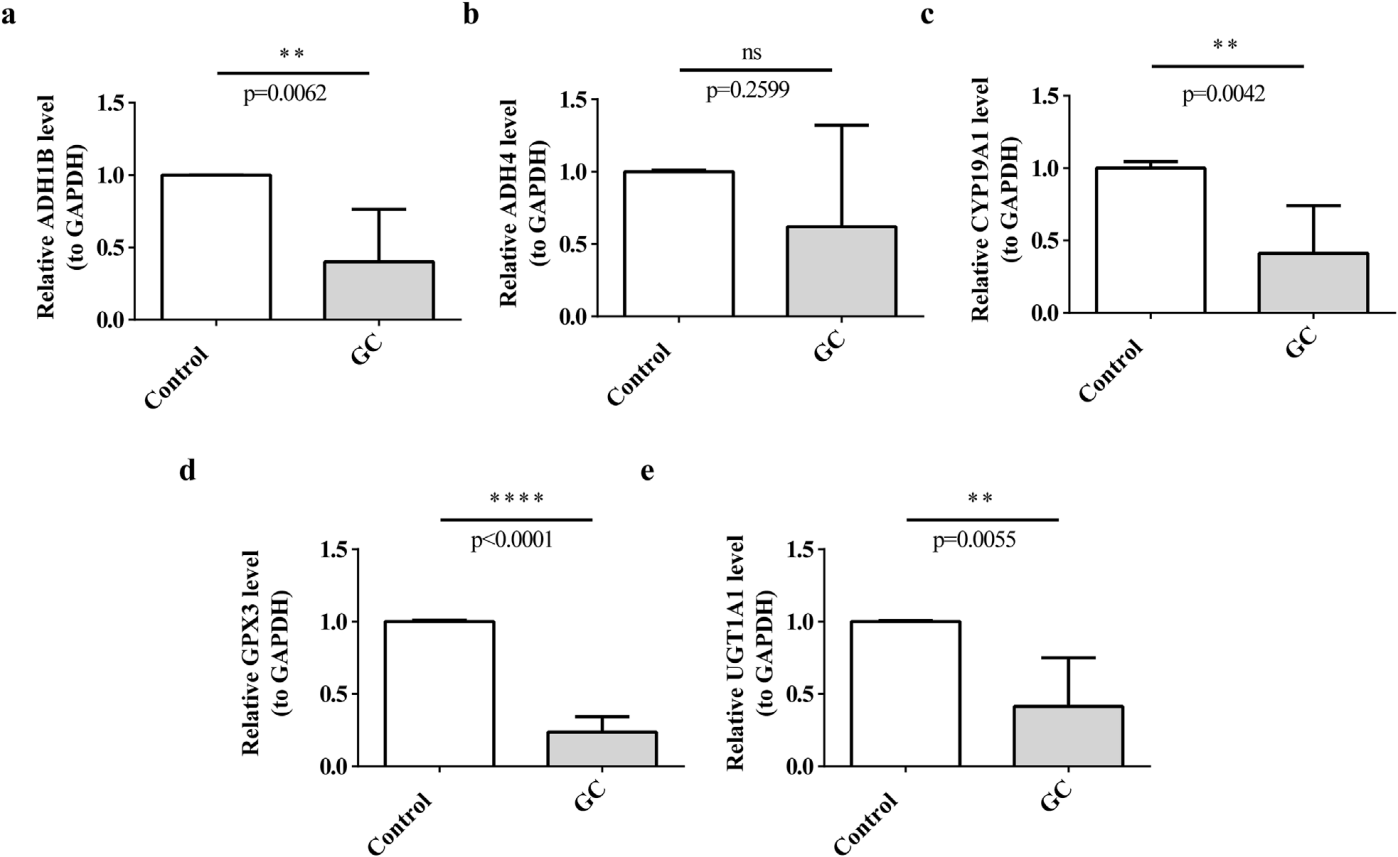
Expression of prognostic genes in clinical samples. **(a)** ADH1B, **(b)** ADH4, **(c)** CYP19A1, **(d)** GPX3, **(e)** UGT1A1.

To explore the expression differences of prognostic genes in the clinic samples, the tumors and para-carcinoma tissues were collected for RT-qPCR. Interestingly, all the prognostic genes except for ADH4 were under-expressed in tumors (P < 0.05), and there was no significant expression difference of ADH4 between the tumors and para-carcinoma tissues (Figures 11a–e).

## 4 Discussion

GC is a leading cause of cancer deaths worldwide, and ADMERGs are increasingly implicated in its progression and therapy. Studying ADMERGs effect on GC can help predict patient prognosis and therapy options. This study found differentially expressed ADMERGs in combination with the TCGA-GC dataset. Five prognostic genes (UGT1A1, ADH4, ADH1B, CYP19A1, and GPX3) were then identified using univariate Cox regression and LASSO regression. Subsequently, Risk scores for GC patients were then obtained based on the prognostic genes and combined with a clinically created nomogram. In recent years, numerous studies have attempted to construct prognostic models for gastric cancer (GC) based on multi-omics features, covering multiple research dimensions such as immune-related genes, m6A modifications, metabolic regulation, and ferroptosis mechanisms. For example, Ma et al. developed a model based on differentially expressed immune-related genes (DEIRGs) to predict overall survival in GC patients and assess immune infiltration levels, though it did not account for the influence of metabolic or drug response pathways (Ma et al., 2024). Similarly, Peng et al. established a prognostic model for the GC tumor microenvironment based on m6A regulator-related genes (Peng et al., 2024). Feng et al. analyzed six tumor-associated metabolic pathways. Wen et al. developed a gastric cancer (GC) model based on ferroptosis-related genes (comprising six genes), which demonstrated prognostic value for patient survival but lacked in-depth investigation into immune phenotypes. Additionally, some studies have constructed models based on different cancer stages. For instance, Liu et al. developed prognostic models for early- and late-stage cancers. Compared to these models, the prognostic model constructed in this study based on five ADME-related genes not only more accurately predicts patient survival by incorporating covariates such as age and clinical stage (T, N, M), thereby providing a basis for developing personalized treatment strategies for gastric cancer (GC) at different stages, but also explores differences in genomic variation, immune microenvironment, and drug sensitivity, investigates the molecular regulatory mechanisms of prognostic genes, and observes the distribution and expression patterns of these genes in single-cell datasets, offering new reference evidence and theoretical support for GC treatment.

UDP glucuronosyltransferase family 1 member A1(UGT1A1) helps detoxify and eliminate toxins from the body and outside sources by glucuronidation. Chemical degradation associated to UGT1A1 expression alterations causes digestive tract cancer. Several studies suggested that UGT1A1 glucuronidases cancer-causing compounds to make them water-soluble and excretable. Chemicals that cause stomach lining cancer are broken down by UGT1A1. And UGT1A1 expression or activity may alter stomach lining cancer-causing material elimination and GC risk. Chemical accumulation from UGT1A1 dysfunction promotes DNA damage and tumor risk, while genetic variations in UGT1A1 can affect GC risk via modifying the enzyme’s chemical elimination (Pereira et al., 2022). Additionally, the detoxification function of UGT1A1 has been confirmed to be closely associated with the risk of digestive system tumors. In this study, it showed significant correlations with PD-L1 and HER2, suggesting its potential important role in immune regulation (Tomono et al., 2023; Sathe et al., 2024). Alcohol dehydrogenase 4 (Class II, ADH4) and alcohol dehydrogenase 1B (Class I, ADH1B), as key enzymes in ethanol metabolism, play a central role in liver ethanol detoxification by catalyzing the oxidation of alcohol to acetaldehyde. Notably, the ADH4-mediated conversion of ethanol to acetaldehyde is an essential step in liver detoxification (PMID: 16801720). However, excessive alcohol consumption significantly increases the risk of gastric cancer (GC), which is closely related to ADH4 (Freedman et al., 2007). The acetaldehyde produced by ADH4 catalysis exhibits high reactivity. On one hand, it can bind to DNA to form adducts, directly inducing gene mutations and compromising the stability of genetic material (Wang et al., 2023). On the other hand, acetaldehyde generated by ADH4 metabolism in gastric tissue can also disrupt the gastric mucosal barrier, creating pathological conditions for carcinogenesis (Nieminen and Salaspuro, 2018). Additionally, polymorphisms in the ADH1B gene significantly influence alcohol metabolism efficiency. Individuals carrying specific variants exhibit reduced acetaldehyde clearance capacity, leading to increased acetaldehyde accumulation during heavy drinking and thereby further elevating the risk of gastric cancer (GC) (Aida et al., 2013; Cui et al., 2009). However, in this study, ADH4 did not show significant downregulation in tumor tissues, suggesting that its expression may be subject to more complex regulatory mechanisms - a phenomenon that has not been adequately discussed in existing research. The aromatase gene cytochrome P450 family 19 subfamily A member 1(CYP19A1) can convert androgens into estrogens, and reproductive tissue growth and function depend on estrogens (Mo et al., 2013). The hormone-sensitive pathways of estrogen signaling are connected to stomach cancer. The estrogen produced by CYP19A1 activates estrogen receptors in gastric tissue, altering cell growth and death. Our study further reveals its low expression in GC tissues, complementing previous literature reporting that its variants may disrupt hormone signaling pathways and thereby promote tumor growth (Frycz et al., 2017). At the level of genetic variation, genetic differences in the CYP19A1 gene can affect aromatase activity, which can affect estrogen levels and gastric epithelial cell hormonal control (Miyoshi et al., 2003). Glutathione peroxidase 3(GPX3), an antioxidant enzyme, reduces hydrogen peroxide and organic hydroperoxides to protect cells. GC tissues show GPX3 downregulation, which increases oxidative stress and tumor development (Chen et al., 2011). Hypermethylation of the GPX3 promoter in GC decreases the output of this protective enzyme, leading to loss of GPX3 activity and causing oxidative damage and malignancy in gastric epithelial cells. Also, an increase in reactive oxygen species (ROS) can damage DNA and lead to cancer, so GPX3 is needed to counteract ROS (Lan et al., 2017). Due to their roles in carcinogen detoxification (UGT1A1), alcohol metabolism and acetaldehyde production (ADH4, ADH1B), hormonal regulation (CYP19A1), and oxidative stress protection (GPX3), these five genes affect GC development. These gene abnormalities can increase GC risk by causing DNA damage, inflammation, metabolic abnormalities and aberrant cell proliferation. These key genes (UGT1A1, ADH4, ADH1B, CYP19A1, and GPX3) are crucial for the construction of our gastric cancer (GC) prognostic model. However, based on the risk scores derived from the prognostic genes and combined with clinical data, the nomogram analysis revealed that the current model’s AUC values ranged between 0.59 and 0.77, failing to exceed 0.8. Nevertheless, existing literature reports that the AUC values of GC-related models vary from 0.560 to 0.989 (Xu et al., 2024), indicating that our newly developed model possesses a certain ability to distinguish between favorable and poor prognoses in patients and holds some clinical guidance significance. However, its accuracy may still be relatively low. Further optimization by incorporating additional clinical information is necessary to enhance its predictive accuracy and generalizability, thereby facilitating more personalized treatment strategies.

Similar to the findings of previous studies, the findings of this research demonstrate the complex correlation between the prognostic gene profile linked to ADME activities and the immune microenvironment in GC (Avolio et al., 2025; Li et al., 2024). The results emphasize notable disparities in the prevalence of immunological and stromal cells among the two indicated risk groups, with specific focus on immune cells such as Effector memory CD8 T cells, Activated B cells, and Central memory T cells. The presence of these differences highlights the potential impact of the immune landscape on the prognosis of patients with GC and the processes that drive tumor growth. The presence of a positive association between the risk score and the quantity of cell types such mast cells, plasmacytoid dendritic cells, and effector memory CD4 T cells indicates that the high-risk group may have a milieu that suppresses the immune system to a greater extent (Lazăr et al., 2018). This could exacerbate the unfavorable outcome reported in these individuals, as such an environment may enhance tumor evasion from immune monitoring and promote cancer development and spread (Yang et al., 2021). The significant variations in the levels of expression of 24 immunological checkpoints, such as the increased expression of BTLA, CD200, and CD40LG in the high-risk group, provide additional evidence that immune evasion mechanisms are more active in patients with higher risk scores. Therefore, utilizing personalized patient risk scores to tailor more effective immunotherapies may represent a promising direction, and future studies could incorporate clinical data from immunotherapy treatments to validate the predictive capability of TIDE scores in real-world therapeutic applications.

Furthermore, the contrasting expression of m6A-RGs in the two risk groups indicates that RNA methylation may have a crucial impact on regulating the immune response and affecting the clinical outcomes of patients with GC. The excessive production of m6A-RGs in the high-risk group may likely cause the disruption of immune-related pathways, leading to the observed changes in immune cell infiltration and checkpoint expression (Zhang et al., 2016). The medication sensitivity assessments offer useful insights into prospective therapy methods for various risk groups. Our research suggested that patients classified as low-risk are more prone to positive responses to medications such as QS11 and ABT.888. These treatments specifically target hypoxia pathways and inflammatory signaling, respectively. On the other hand, the high-risk group was more sensitive to drugs such as DMOG and VX.702. The Spearman association study provides more evidence, indicating a positive correlation between the risk score and sensitivity to BIBW2992, which is an EGFR inhibitor. Conversely, there is a negative correlation between the risk score and the drugs DMOG, Imatinib, AP.24534, and VX.702. The findings indicate that the prognostic gene signature not only has the ability to predict patient outcomes, but also has the potential to inform treatment decisions in clinical practice. Patients categorized as high-risk may get advantages from treatments that specifically target immunological checkpoints or EGFR signaling, and individuals in the high-risk group may also respond better to medicines that target hypoxia and inflammatory pathways. ESCC is characterized by mutations in the p53 gene, which enhance the production of the AGAP1 protein. This protein, in turn, increases the synthesis of exosomes, leading to the accelerated growth and spread of cancer cells (Feng et al., 2023). According to a study, QS11 has the ability to hinder the activity of AGAP1, which in turn reduces the growth and spread of ESCC cells. Furthermore, research has identified somatic (P53) mutations in GC as well (Han et al., 2024). The study found that low-risk patients showed considerable enrichment in pathways such as DNA replication and nucleotide excision repair. It is hypothesized that patients with a low risk profile may have a higher likelihood of seeing positive outcomes from gene- and protein-level therapy.

The integration of differential expression analysis, mRNA-miRNA-lncRNA interaction prediction, and scRNA-seq provides a comprehensive view of the molecular and cellular heterogeneity in GC (Li et al., 2025; Wan et al., 2015). Significant differences in miRNAs (e.g., mir590, mir7152, mir4420, mir5000) were identified between the high-risk and low-risk gastric cancer (GC) groups. These miRNAs exhibited significantly upregulated expression in the high-risk group, suggesting their potential role in suppressing oncogenic pathways to inhibit GC tumor growth, which aligns with existing literature. Similarly, the lncRNA (MIR100HG) was also markedly upregulated in the high-risk group. Previous studies have demonstrated that MIR100HG serves as a reliable prognostic biomarker associated with GC cell proliferation, migration, and invasion. The differentially expressed miRNAs and lncRNAs uncovered in this study may play pivotal roles in GC progression. They hold promise as prognostic markers and could provide novel therapeutic avenues for GC treatment.

ScRNA-seq data from GC tissues showed significant cell diversity using Seurat R package. ScRNA-seq data also showed significant differences in the expression levels of key prognostic genes such ADH1B, ADH4, CYP19A1, and GPX3 across cell types. The lower expression of these genes in tumor tissues suggests a role in GC onset and progression. For instance, decreased ADH1B and ADH4 expression in tumor cells can lead to acetaldehyde accumulation, which can promote cancer, DNA damage, and tumor growth. Decreased GPX3 may increase oxidative stress, which promotes gastric epithelial cell malignancy (Ren et al., 2021).

GC development’s molecular mechanisms can be understood by studying miRNA-lncRNA interactions and single-cell data. The identified miRNAs, lncRNAs, and target genes may be GC biomarkers. Additionally, they could be targeted for new therapeutic methods. Customized treatment techniques that account for tumors' diverse cell and molecular profiles are crucial because GC tissues contain distinct types of cells. ADME-related prognostic genes vary in expression in different cell types, suggesting that they can affect the metabolic milieu of the tumor microenvironment (TME) and targeted therapeutic efficacy (Gu et al., 2015).

Regarding the limitations of current GC diagnostic methods (such as gastroscopy and tumor marker detection) in terms of sensitivity and specificity, this study identified five ADME-related prognostic genes (UGT1A1, ADH4, ADH1B, CYP19A1, and GPX3) that exhibit unique expression patterns in tumor tissues (UGT1A1, ADH1B, CYP19A1, and GPX3 show significant downregulation). These findings hold potential for developing new tools and strategies to improve GC diagnosis and treatment. First, a multiplex gene quantification assay based on minimally invasive samples (peripheral blood, gastric juice, exfoliated cells) could be developed for early screening of high-risk populations (e.g., individuals with family history or chronic *H. pylori* infection). This tool could compensate for the limitations of gastroscopy, reduce missed diagnoses, and improve early detection rates. Second, detecting ADME gene expression (e.g., downregulation of UGT1A1, ADH1B, GPX3) in gastroscopically suspicious lesions could enhance diagnostic accuracy through combined assessment, guide further examinations, and reduce false-negative results. Additionally, an ADME risk score could effectively stratify GC patients into high-risk and low-risk groups. Significant differences were observed between the two groups regarding immune microenvironment, drug sensitivity, and prognosis. The high-risk group (with poorer prognosis) requires aggressive treatment strategies, including neoadjuvant chemotherapy combined with targeted therapy (e.g., EGFR inhibitors), radical surgery, and adjuvant therapy, to reduce recurrence risk. In contrast, the low-risk group (with better prognosis) should avoid overtreatment, with surgical intervention or low-toxicity adjuvant chemotherapy being preferred options, along with intensified postoperative surveillance. These findings provide valuable guidance for developing precision medicine strategies.

Due to the relatively limited sample size in the training, validation and scRNA-seq datasets, particularly the scarcity of clinically normal tissue samples—our study may be susceptible to batch effects or population bias. This constraint likely hindered the model’s ability to fully capture the comprehensive features and distribution patterns of the data, partially compromising the stability and reliability of the results (AUC >0.6 but <0.8) and limiting the generalizability of findings to broader populations. Similarly, the inconsistent results between RT-qPCR and single-cell data are likely attributable to insufficient sample size and sample heterogeneity. Furthermore, insufficient sample resources led to an extremely small clinical validation cohort (n = 5 for tumor/adjacent tissues), and the regulatory mechanisms of the ceRNA network relied solely on bioinformatic predictions without experimental validation, weakening the robustness of conclusions. Moving forward, we will further investigate the differential activity of these genes in specific cell populations. This will involve experimental validation of the ceRNA network’s regulatory mechanisms through approaches such as RNA interference (RNAi), overexpression assays, constructing animal models, and gene knockout experiments. These studies aim to elucidate the precise role of the ceRNA network in gastric cancer (GC) initiation and progression, as well as its potential crosstalk with other regulatory pathways. Finally, we will attempt to obtain additional single-cell sample data for analysis and collect extra clinical samples to validate our research findings via qPCR. Meanwhile, we will specifically measure the relationship between TIDE scores in the high-risk group and actual immune therapy responses to further verify the predictive value of TIDE scores. These efforts are expected to provide more reliable and actionable insights for improving GC treatment strategies.

This work concludes by offering a thorough investigation of the correlation between ADME-related prognostic genes and the immunological microenvironment in GC. The recognition of unique immunological profiles and medication sensitivities linked to various risk groups emphasizes the possibility of tailored treatment approaches in GC. Additional investigation is necessary to examine the fundamental mechanisms that cause these connections and to confirm these discoveries in medical environments, with the ultimate objective of enhancing patient outcomes through customized therapy methods. Our research helps us grasp GC’s complex molecular and cellular networks. The found miRNAs, lncRNAs, and prognostic genes offer promising potential for further research and therapeutic applications, such as targeted medicines and personalized GC treatment.

## 5 Conclusion

This study effectively discovered predictive genes associated to ADME in GC by thorough bioinformatics analysis of data from the TCGA and GEO databases. The results provide useful knowledge on the genetic basis of GC and emphasize the potential of these genes as biomarkers for the detection, prediction of outcome, and targets for treatment. Through the integration of differential expression analysis, miRNA-lncRNA interaction prediction, and single-cell RNA sequencing data, we have discovered noteworthy connections between these prognostic genes, the tumor microenvironment, and cellular heterogeneity in GC.

The identified genes and their regulatory networks serve as a new reference framework for future study in GC, namely in comprehending the disease’s course and in formulating tailored treatment methods. In order to further understand how these prognostic genes, contribute to the development of GC, it is necessary to conduct additional experimental research to uncover the exact mechanisms involved. Moreover, the practical implementation of these discoveries, which involves creating specific treatments, shows potential for enhancing the identification and management of patients with GC.

## Data Availability

The datasets analyzed for this study can be found in the TCGA database and GEO database [http://portal.gdc.cancer.gov/ and https://www.ncbi.nlm.nih.gov/geo/], accession number TCGA-GA, GSE62254 and GSE13911.
