# Supplementary material for: Construction of a novel prognostic model for gastric cancer based on pharmacokinetics-related genes and comprehensive prognostic analysis
Source: Front Genet. 2025 Sep 15;16:1541401. doi: 10.3389/fgene.2025.1541401 (PMC12477026; doi:10.3389/fgene.2025.1541401)
Supplement: Supplementary file 1 [file DataSheet2.pdf]

GeneSymbol	baseMean	log2FoldChange	lfcSE	stat
MIR590	18.9639572466174	1.0595784792753	0.157557079546742	6.72504518567803
MIR7152	1.20715415879679	2.0899361511539	0.312296925439916	6.69214449745195
MIR4420	3.09510267587559	1.00885888974985	0.21756547612551	4.63703574535813
MIR5000	2.07093608365834	1.03306670791756	0.267852463957356	3.85684974726249

pvalue	padj	change
1.75537960155317e-11	7.75230475867929e-09	UP
2.19923539253313e-11	7.75230475867929e-09	UP
3.53441307351913e-06	0.000177982944059356	UP
0.000114857714217636	0.00249152887764411	UP
