# Supplementary figures and images for: Construction of a novel prognostic model for gastric cancer based on pharmacokinetics-related genes and comprehensive prognostic analysis
Source: Front Genet. 2025 Sep 15;16:1541401. doi: 10.3389/fgene.2025.1541401 (PMC12477026; doi:10.3389/fgene.2025.1541401)

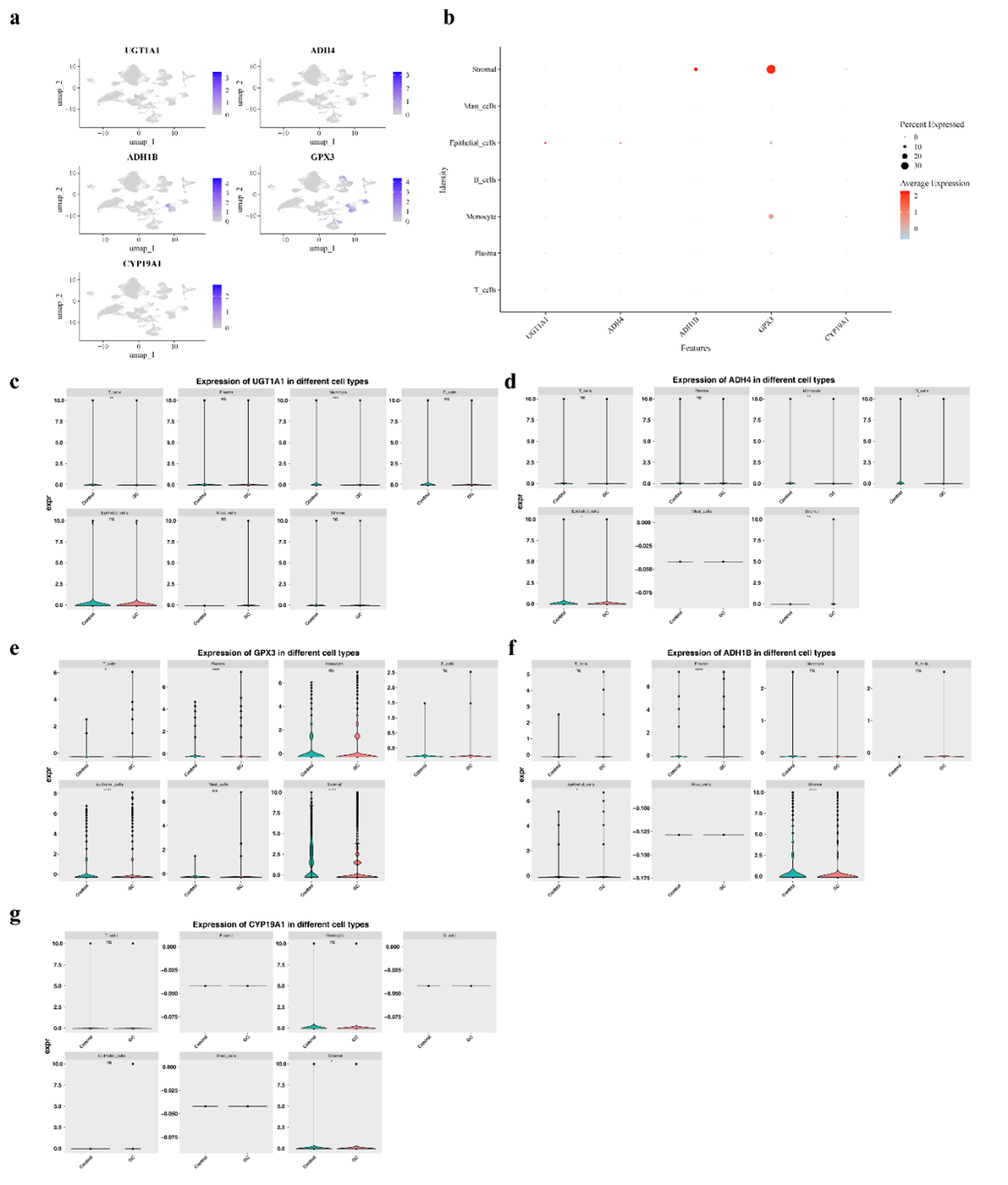

Supplement: Supplementary file 2 [file Image6.tif]

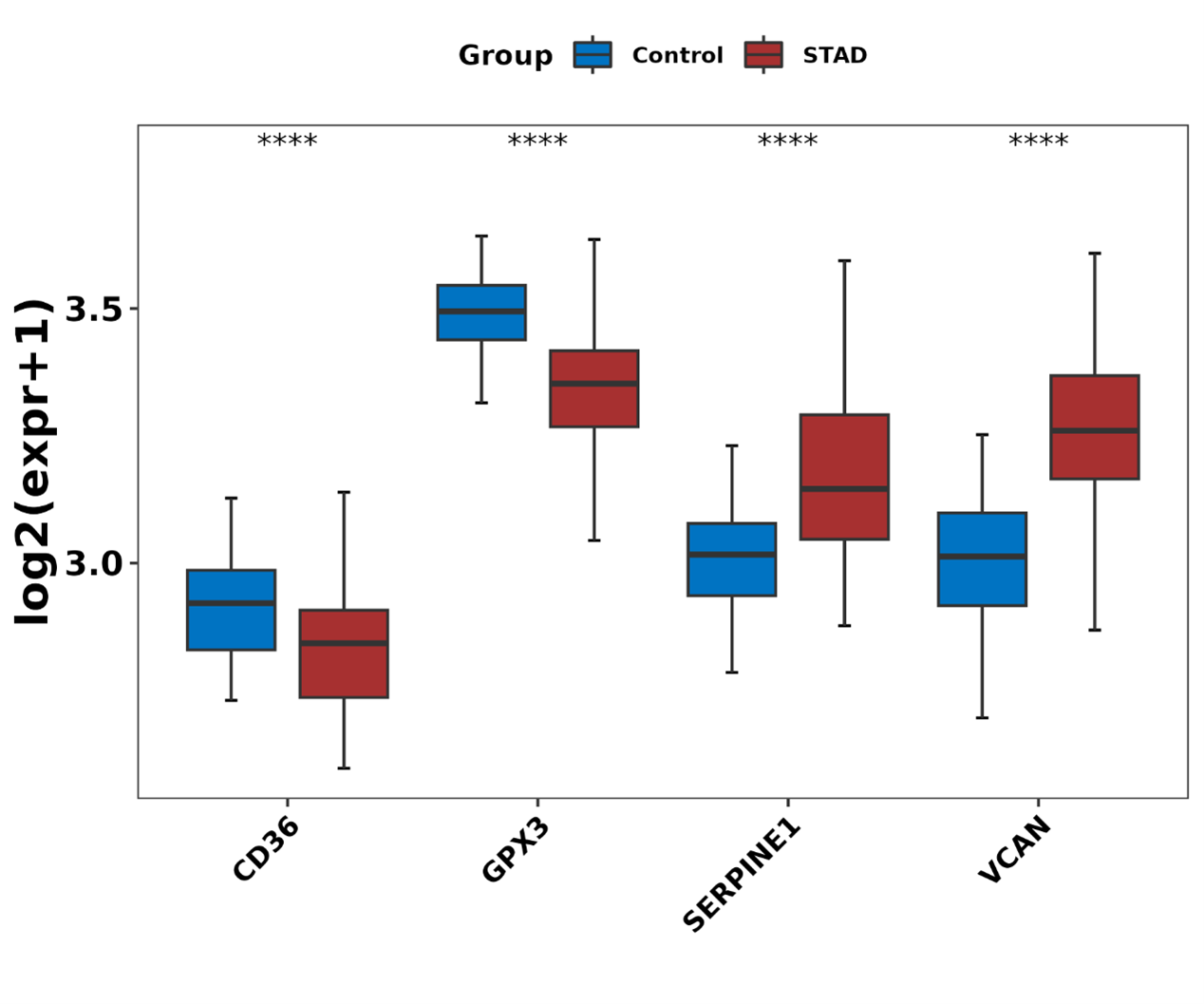

Supplement: Supplementary file 4 [file Image4.png]

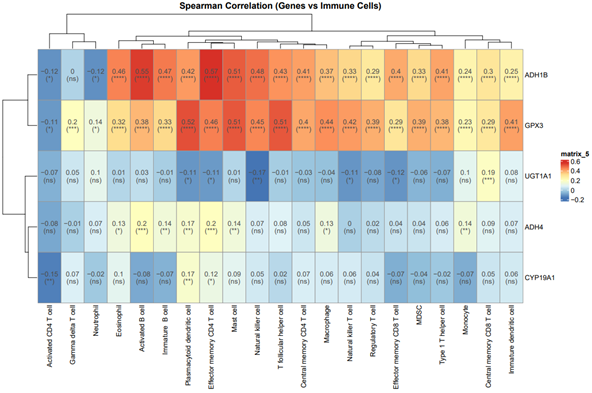

Supplement: Supplementary file 6 [file Image2.png]

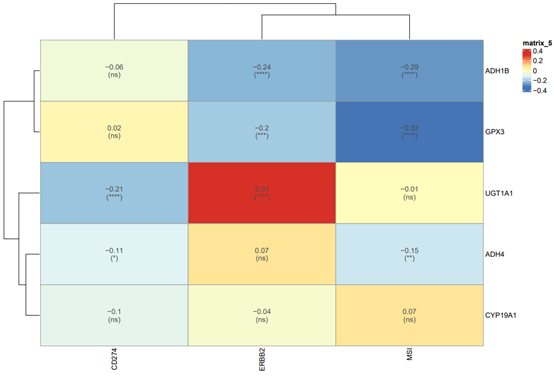

Supplement: Supplementary file 7 [file Image1.png]

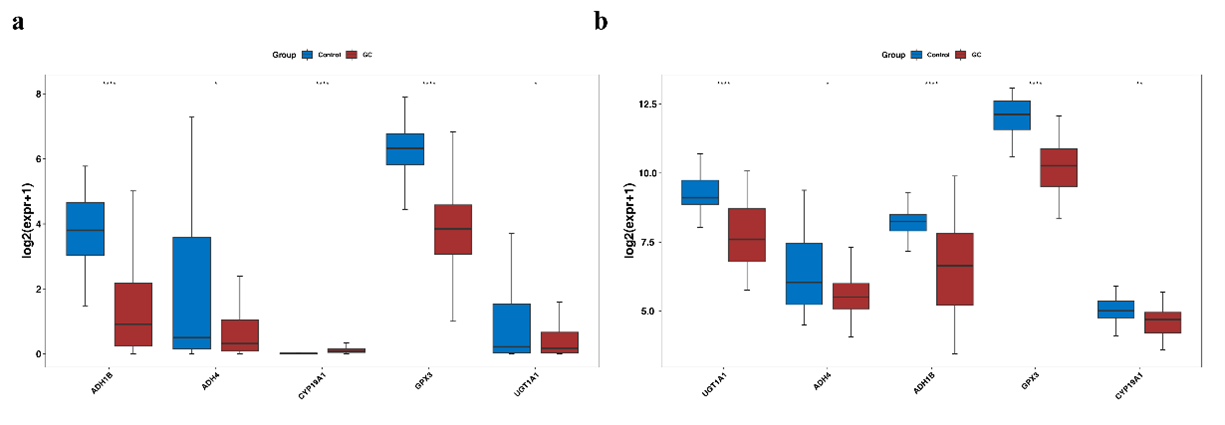

Supplement: Supplementary file 8 [file Image5.tif]

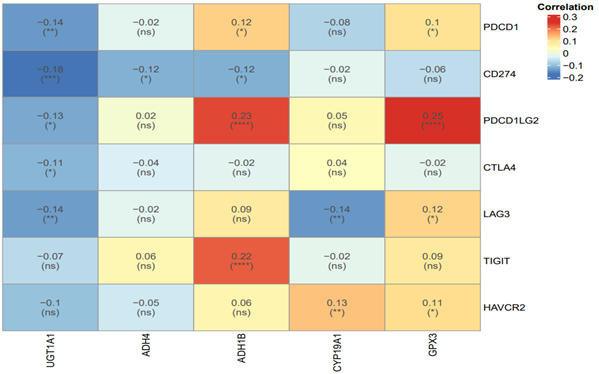

Supplement: Supplementary file 9 [file Image3.png]
