# Supplementary material for: Construction of a novel prognostic model for gastric cancer based on pharmacokinetics-related genes and comprehensive prognostic analysis
Source: Front Genet. 2025 Sep 15;16:1541401. doi: 10.3389/fgene.2025.1541401 (PMC12477026; doi:10.3389/fgene.2025.1541401)
Supplement: Supplementary file 3 [file DataSheet3.pdf]

GeneSymbol	baseMean	log2FoldChange	lfcSE	stat
MIR100HG	18.873948609302	1.08455009560282	0.167305317087489	6.48246041717652

pvalue	padj	change
9.02387636389176e-11	1.52527899404808e-08	UP
