# Supplementary material for: Construction of a novel prognostic model for gastric cancer based on pharmacokinetics-related genes and comprehensive prognostic analysis
Source: Front Genet. 2025 Sep 15;16:1541401. doi: 10.3389/fgene.2025.1541401 (PMC12477026; doi:10.3389/fgene.2025.1541401)
Supplement: Supplementary file 5 [file DataSheet1.pdf]

Primer name	sequence (5'-3')
ADH1B-F	GACTCACAGTCTGCTGGTGG
ADH1B-R	GCATTTGATTACTTTTCCTGCTGT
ADH4-F	TTCAACTGGCTATGGGGCTG
ADH4-R	CCTAGGCCAAAGACAGCACA
CYP19A1-F	AGCCCATCAAACCAGGACTC
CYP19A1-R	CATCCACAGGAATCTGCCGT
GPX3-F	AGAAGTCGAAGATGGACTGCC
GPX3-R	GGGAAAGCCCAGAATGACCA
UGT1A1-F	ACTGTTGATCCCAGTGGATGG
UGT1A1-R	TCCTCCCTTTGGAATGGCAC
GAPDH-F	CGAAGGTGGAGTCAACGGATTT
GAPDH-R	ATGGGTGGAATCATATTGGAAC
